# Sleeve Gastrectomy-Induced Weight Loss Increases Insulin Clearance in Obese Mice

**DOI:** 10.3390/ijms24021729

**Published:** 2023-01-15

**Authors:** Gabriela Moreira Soares, Luana Emanuelly Sinhori Lopes, Sandra Lucinei Balbo, Carine Marmentini, Gabriela Alves Bronczek, Mirian Ayumi Kurauti, Maria Lúcia Bonfleur, Licio Augusto Velloso, Everardo Magalhães Carneiro, Antonio Carlos Boschero, José Maria Costa-Júnior

**Affiliations:** 1 Obesity and Comorbidities Research Center, Institute of Biology, University of Campinas (UNICAMP), Campinas 13083-864, Brazil; 2 Laboratory of Endocrine Physiology and Metabolism, Biological Sciences and Health Center, Western Paraná State University (UNIOESTE), Cascavel 85819-110, Brazil; 3 Center for Diabetes Research, Division of Endocrinology, Erasmus Hospital, Universite Libre de Bruxelles (ULB), 1070 Brussels, Belgium

**Keywords:** insulin-degrading enzyme, hepatic insulin clearance, hyperinsulinemia, bariatric surgery, obesity

## Abstract

Sleeve gastrectomy (SG) successfully recovers metabolic homeostasis in obese humans and rodents while also resulting in the normalization of insulin sensitivity and insulinemia. Reduced insulin levels have been attributed to lower insulin secretion and increased insulin clearance in individuals submitted to SG. Insulin degradation mainly occurs in the liver in a process controlled, at least in part, by the insulin-degrading enzyme (IDE). However, research has yet to explore whether liver IDE expression or activity is altered after SG surgery. In this study, C57BL/6 mice were fed a chow (CTL) or high-fat diet (HFD) for 10 weeks. Afterward, the HFD mice were randomly assigned to two groups: sham-surgical (HFD-SHAM) and SG-surgical (HFD-SG). Here, we confirmed that SG improves glucose–insulin homeostasis in obese mice. Additionally, SG reduced insulinemia by reducing insulin secretion, assessed by the analysis of plasmatic C-peptide content, and increasing insulin clearance, which was evaluated through the calculation of the plasmatic C-peptide:insulin ratio. Although no changes in hepatic IDE activity were observed, IDE expression was higher in the liver of HFD-SG compared with HFD-SHAM mice. These results indicate that SG may be helpful to counteract obesity-induced hyperinsulinemia by increasing insulin clearance, likely through enhanced liver IDE expression.

## 1. Introduction

Bariatric surgery is an effective measure to counteract obesity complications, i.e., glucose intolerance, insulin resistance, and hyperinsulinemia [1,2,3,4,5]. The reduction in the plasmatic insulin levels after Roux-en-Y gastric bypass surgery (RYGB) has largely been linked to augmented insulin clearance rather than reduced insulin secretion in humans [6,7,8,9,10]. An increase in human insulin clearance after sleeve gastrectomy (SG) also has been reported [7,11].

Around 80% of insulin is degraded in the hepatocytes during its first liver passage, and the insulin-degrading enzyme (IDE) seems to be crucial for this process [12,13]. Indeed, IDE-KO mice develop insulin resistance associated with dramatic hyperinsulinemia [14].

Furthermore, insulin-resistant obese mice fed a high-fat diet develop a reduction in insulin clearance associated with reduced liver IDE expression and activity. The recovery of insulin sensitivity through the performance of physical exercise or bile acid TUDCA administration restored insulin clearance and liver IDE expression in those mice [15,16].

The majority of the studies exploring insulin clearance after bariatric surgery were performed in humans, impeding the analysis of the possible involvement of liver IDE in this context. Therefore, we sought to verify whether the mouse model of SG mirrors the outcomes related to insulin clearance observed in humans and whether this outcome is regulated by liver IDE expression and activity.

Here, we demonstrated that SG decreases insulinemia in HFD-SG mice, a phenomenon associated with increased insulin clearance and liver IDE expression. Together, these results provide evidence that SG affects hepatic insulin clearance and may be helpful to counteract obesity-induced hyperinsulinemia.

## 2. Results

### 2.1. HFD Increases Body Weight Gain and Impairs Glucose Tolerance and Insulin Sensitivity in Mice

**Figure 1:**
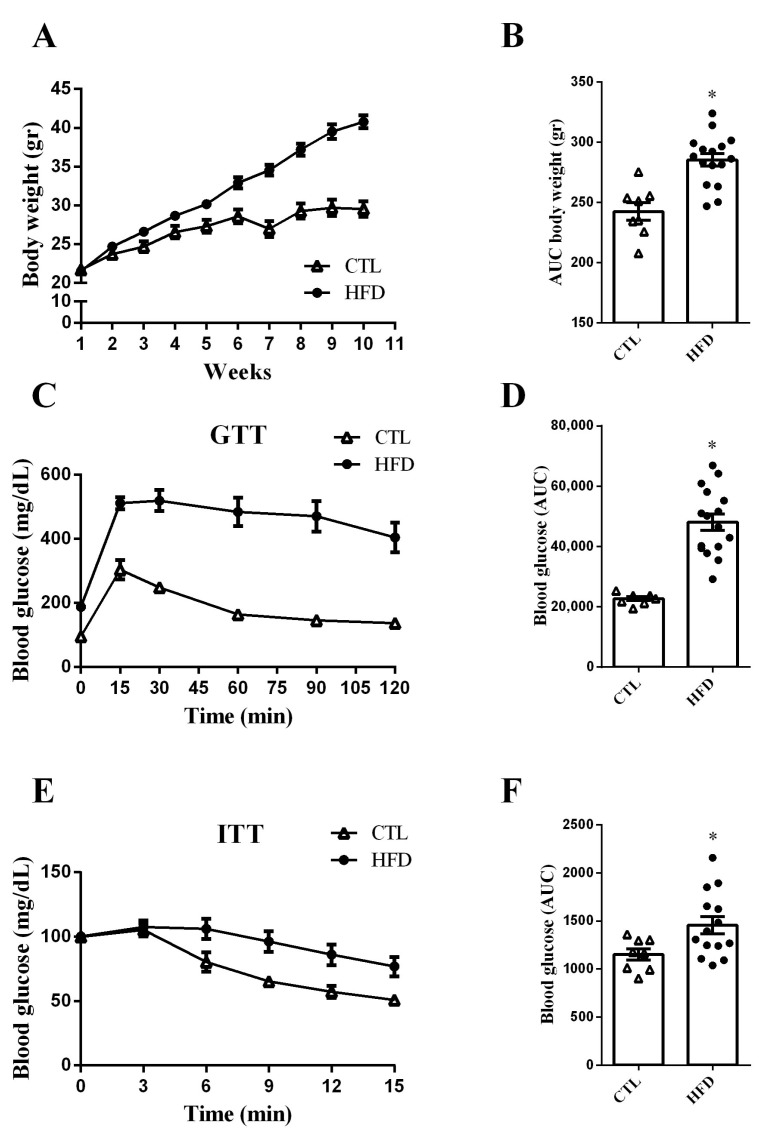
HFD increases body weight gain and impairs glucose tolerance and insulin sensitivity in mice. Body weight over the course of the experimental period (**A**) and its respective area under the curve (AUC) (**B**) of CTL and HFD mice (n = 8–16). Blood glucose of CTL and HFD mice during the intraperitoneal glucose tolerance test (ipGTT) (**C**) (n = 8–16). The area under the curve (AUC) of total blood glucose concentration of CTL and HFD mice during ipGTT (**D**) (n = 8–16). Blood glucose of CTL and HFD during the intraperitoneal insulin tolerance test (ipITT) (**E**) (n = 8–14). The area under the curve (AUC) of total blood glucose concentration of CTL and HFD during ipITT (**F**) (n = 8–14). Data are the mean ± SEM. * *p* ≤ 0.05 (Student’s *t*-test).

Firstly, we evaluated the body weight gain of mice receiving a high-fat diet (HFD) over the course of ten weeks. As expected, HFD mice displayed higher body weight gain compared with control (CTL) mice (Figure 1A), as confirmed by the area under the curve (AUC) (Figure 1B).

Next, to investigate the effects of the HFD on glucose homeostasis, we performed intraperitoneal glucose and insulin tolerance tests (ipGTT and ipITT). We observed that HFD mice presented impaired glucose tolerance, as determined by the higher AUC of blood glucose during ipGTT (Figure 1C,D). Furthermore, HFD mice also displayed insulin resistance (Figure 1E,F).

### 2.2. SG Reduces Body Weight Gain and Improves Glucose Tolerance and Insulin Sensitivity in HFD-SG Mice

**Figure 2:**
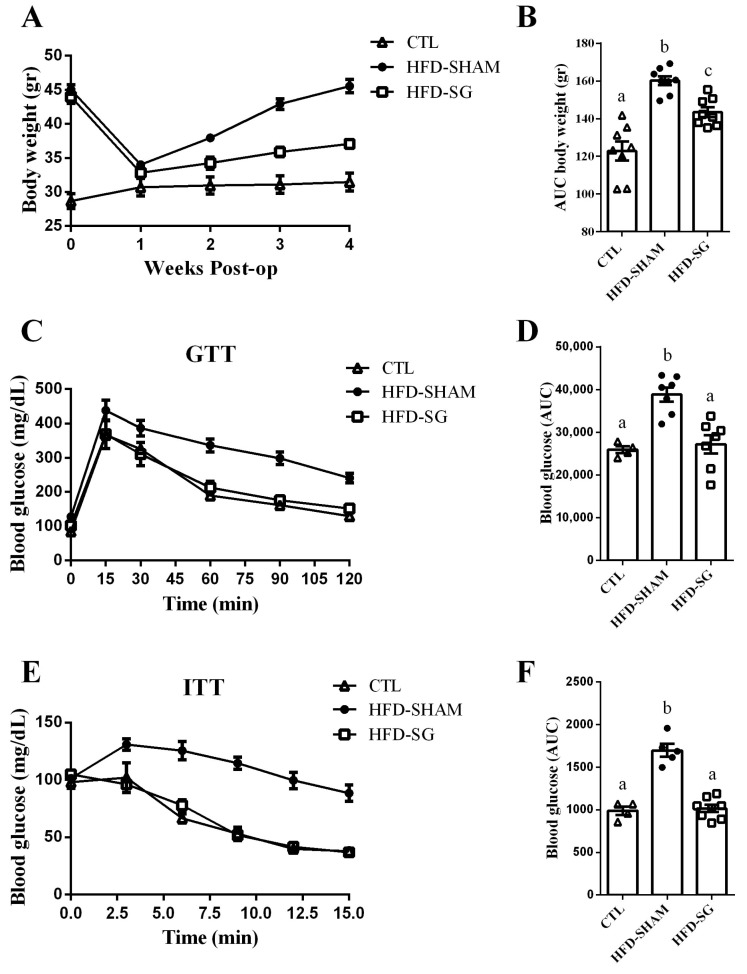
SG reduces body weight gain and improves glucose tolerance and insulin sensitivity in HFD-SG mice. Postoperative body weight (**A**) and its respective area under the curve (AUC) (**B**) of CTL, HFD-SHAM, and HFD-SG mice (n = 8). Blood glucose of CTL, HFD-SHAM, and HFD-SG mice during the intraperitoneal glucose tolerance test (ipGTT) (**C**) (n = 4–7). The area under the curve (AUC) of total blood glucose concentration of CTL, HFD-SHAM, and HFD-SG mice during ipGTT (**D**) (n = 4–7). Blood glucose of CTL, HFD-SHAM, and HFD-SG mice during the intraperitoneal insulin tolerance test (ipITT) (**E**) (n = 4–8). The area under the curve (AUC) of total blood glucose concentration of CTL, HFD-SHAM, and HFD-SG mice during ipITT (**F**) (n = 4–8). Data are the mean ± SEM. Different letters indicate statistical differences between groups, *p* ≤ 0.05 (one-way ANOVA or Kruskal–Wallis test).

Once HFD mice presented overweight with glucose intolerance and insulin resistance, they were either submitted or not to sleeve gastrectomy (HFD-SG or HFD-SHAM, respectively). After that, body weight was measured once a week following postoperative care. As observed in Figure 2A, before the surgical procedure, HFD-SHAM and HFD-SG mice presented similar body weights, which were higher than CTL mice. In addition, due to the postoperative care, both HFD-SHAM and HFD-SG mice lost weight, achieving values close to those of the CTL mice (week 1) (Figure 2A). In the subsequent weeks, HFD-SHAM mice displayed increased body weight gain, recovering similar values to those of the preoperative period (Figure 2A), whereas body weight gain in HFD-SG mice was less pronounced, confirming the efficacy of the SG (Figure 2B). To assess the effect of SG on glucose homeostasis in HFD mice, postoperative ipGTT and ipITT were performed, with HFD-SG mice presenting improved glucose (Figure 2C,D) and insulin (Figure 2E,F) tolerance compared with HFD-SHAM mice.

### 2.3. SG Reduces Fasting and Fed Insulin and C-Peptide Levels While Increasing Hepatic Insulin Clearance in HFD-SG Mice

**Figure 3:**
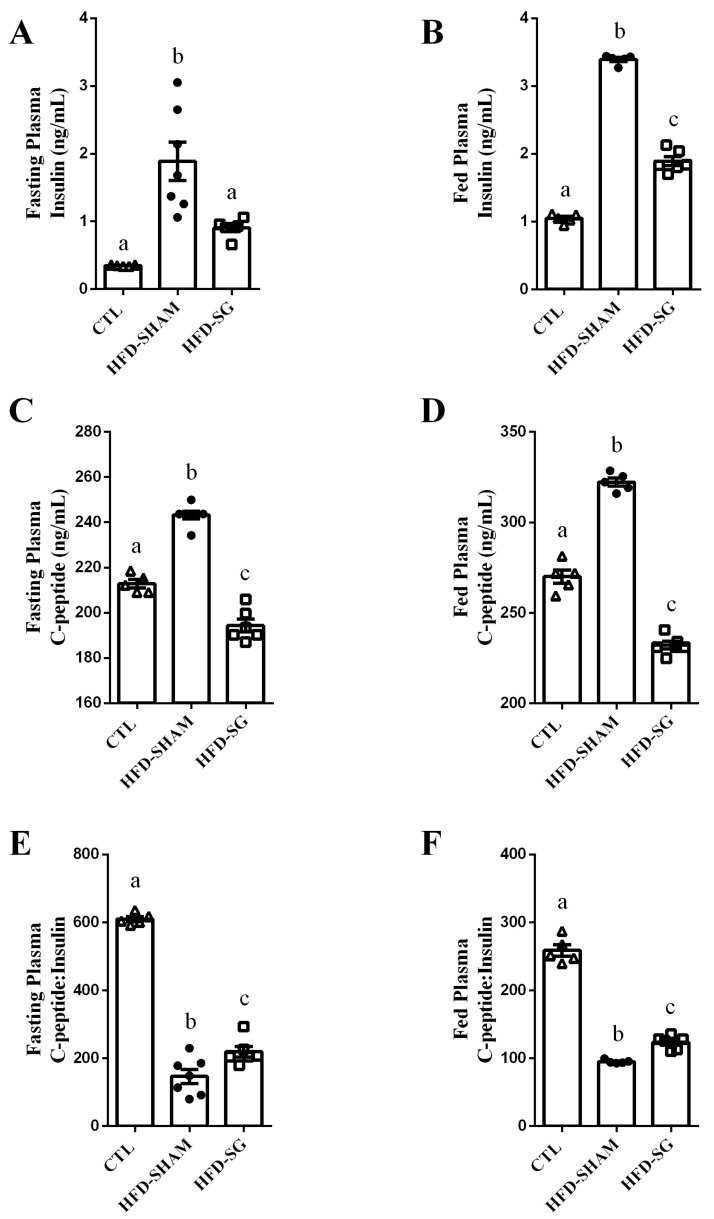
SG decreases fasting and fed insulin and C-peptide levels while increasing hepatic insulin clearance in HFD-SG mice. Plasma insulin in fasting (**A**) and fed (**B**) state of CTL, HFD-SHAM, and HFD-SG mice (n = 5–7). Plasma C-peptide in fasting (**C**) and fed (**D**) state of CTL, HFD-SHAM, and HFD-SG mice (n = 5–7). The C-peptide:insulin ratio in fasting (**E**) and fed (**F**) state of CTL, HFD-SHAM, and HFD-SG mice (n = 5–7). Data are the mean ± SEM. Different letters indicate statistical differences between groups, *p* ≤ 0.05 (one-way ANOVA or Kruskal–Wallis test).

Evidence suggests that glucose homeostasis improves after bariatric operations due to, at least in part, an increase in insulin clearance. Thus, we evaluated insulin clearance in obese mice after performing SG operation by calculating the C-peptide:insulin ratio in fasting and fed states. The HFD-SHAM mice displayed higher plasma insulin compared with CLT mice in fasting (Figure 3A) and fed states (Figure 3B). However, HFD-SG mice presented lower plasma insulin compared with HFD-SHAM mice in both fasting (Figure 3A) and fed (Figure 3B) states. When we evaluated plasma C-peptide, HFD-SHAM mice showed higher values compared with CTL mice (Figure 3C,D). In addition, HFD-SG mice presented lower plasma C-peptide levels than HFD-SHAM mice and even lower levels than CTL mice in fasting (Figure 3C) and fed states (Figure 3D). These results suggest an increase in insulin clearance, confirmed by the increased C-peptide:insulin ratio in the HFD-SG mice compared with HFD-SHAM mice (Figure 3E,F).

### 2.4. SG Increases Hepatic IDE Expression but Not IDE Activity in HFD-SG Mice

**Figure 4:**
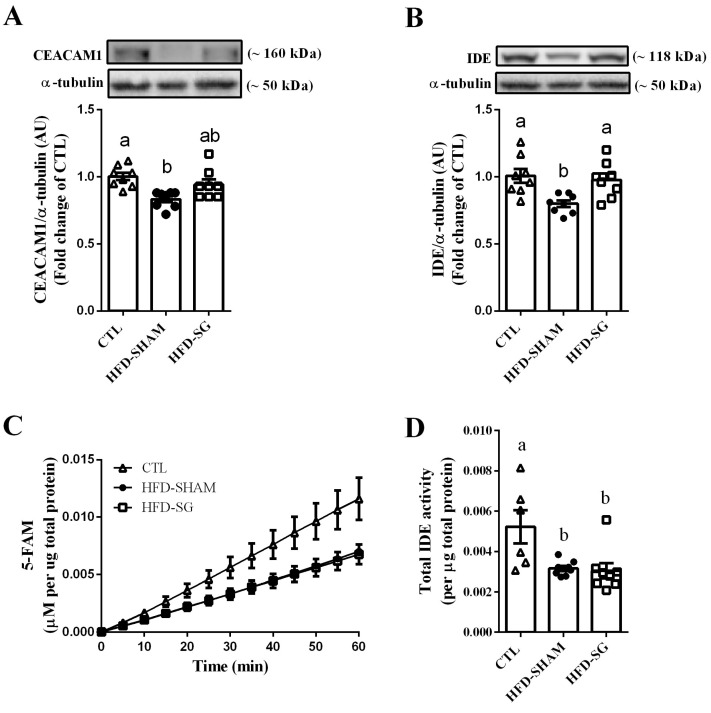
SG increases hepatic IDE expression but not IDE activity in HFD-SG mice. Protein expression of CEACAM1 (**A**) and IDE (**B**) in the liver of CTL, HFD-SHAM, and HFD-SG mice (n = 8). Kinetic of IDE activity assay (**C**) and total IDE activity (**D**) in the liver of CTL, HFD-SHAM, and HFD-SG mice (n = 6–8). Fluorescent intensity at Ex/Em = 490/520 nm was recorded every 5 min for 60 min. 5-Carboxyfluorescein (5-FAM) concentration was calculated using a standard curve and normalized per µg of total protein. Data are the mean ± SEM. Different letters indicate statistical differences between groups, *p* ≤ 0.05 (one-way ANOVA or Kruskal–Wallis test).

To investigate the molecular mechanism by which SG increases hepatic insulin clearance, we evaluated the expression of proteins involved in this process. Expression of carcinoembryonic antigen-related cell adhesion molecule 1 (CEACAM1), the transmembrane protein involved with the endocytosis of the insulin-insulin receptor (IR) complex, was decreased in the liver of HFD-SHAM mice compared with CTL mice (Figure 4A). IDE, the major enzyme responsible for insulin clearance, also experienced reduced expression (Figure 4B) and activity (Figure 4C,D) in the liver of HFD-SHAM mice compared with CTL mice. Furthermore, while SG surgery did not alter hepatic CEACAM1 expression (Figure 4A) and IDE activity (Figure 4C,D), the surgical procedure did increase IDE hepatic expression (Figure 4B) in the HFD-SG mice, in accordance with the higher insulin clearance observed in these mice.

## 3. Discussion

Here, we demonstrated that our SG mice model exhibited beneficial effects on glucose homeostasis and body weight, in agreement with previous studies [17,18,19,20]. In addition, we observed that HFD-SG mice presented increased insulin clearance, as observed in humans [7,11]. These results support previous findings, indicating that SG in mice is an experimental model that accurately mimics the effects observed in humans.

Hyperinsulinemia may precede the insulin resistance observed in obese rodents and humans [21,22]. It occurs because the chronic maintenance of the hyperinsulinemic state overstimulates the insulin receptors, which may cause downregulation of the receptors, thus compromising the insulin signaling pathway [23,24,25]. Along with increased insulin secretion, reduced insulin clearance is also a critical factor in hyperinsulinemia development. In fact, ethnic groups presenting a reduction in insulin clearance present a higher risk of developing type 2 diabetes mellitus [26,27,28]. Obese humans and rodents also have shown decreased insulin clearance [15,29]. Therefore, improving insulin clearance may be crucial to the attenuation of insulin resistance and glucose intolerance.

To measure insulin clearance, we assessed the plasma concentration of C-peptide and insulin in the fasting state and after re-feeding and calculated the ratio between these molecules [30]. Insulin and C-peptide are co-secreted by the pancreatic β cells (ratio 1:1); however, C-peptide has a longer half-life than insulin [15,31,32]. Thus, a decrease in the C-peptide:insulin ratio indicates a reduction in insulin clearance. As expected, insulin clearance was reduced in HFD-SHAM mice compared with controls (Figure 3E,F). The SG surgery partially recovered insulin clearance, as judged by the elevation of the C-peptide:insulin ratio of HFD-SG mice compared with HFD-SHAM mice (Figure 3E,F). These findings are in agreement with studies performed on humans [7,11].

The liver is the most important site for insulin degradation through insulin clearance. This process is initiated when insulin binds to its receptor. After IR is activated by insulin, CEACAM1—an important protein that promotes receptor-mediated insulin internalization—is activated and associated with the insulin–IR complex, triggering the endocytosis process. Then, insulin is cleaved by the major enzyme responsible for its degradation, IDE [12,13].

It has been shown that obesity decreases CEACAM1 and IDE expression [15,33]. Hepatic cell lines exposed to palmitic acid and pro-inflammatory cytokines display decreased IDE gene expression and protein content [34,35]. This indicates that the elevated free fatty acids and pro-inflammatory cytokines present in obese individuals could be involved with the obesity-induced reduction in insulin clearance.

As expected, hepatic CEACAM1 and IDE expression (Figure 4A,B), as well as IDE activity (Figure 4C,D), were reduced in HFD-SHAM mice, supporting the lower insulin clearance in this group. Although SG did not alter CEACAM1 expression and IDE activity (Figure 4A,C,D), it restored IDE hepatic protein expression in HFD-SG mice to levels similar to CTL mice (Figure 4B), explaining at least in part the reestablishment of insulin clearance in these mice.

Additionally, IDE degrades insulin in the endosomes that are formed after insulin binds to IR [12,13,36]. Considering that insulin sensitivity was improved after the SG surgery, it is plausible to speculate that more insulin is present in the endosomes, increasing the capacity of IDE to interact with its substrate and augmenting insulin degradation without altering its activity.

The mechanism underlying enhanced IDE protein content by SG still needs to be clarified, but some hypotheses can be proposed. Bariatric surgeries increase the expression of AMP-activated protein kinase (AMPK) [37] and nuclear respiratory factor 1 (NRF-1) [38]. AMPK induces IDE protein content in neuronal tissue [39], and NRF-1 is a transcription factor related to IDE transcription activation [40]. Bariatric surgeries also stimulate the release of endogenous molecules, such as the fibroblast growth factor (FGF)15/19, which presents several physiological benefits [41], with some of them being orchestrated by the FGF15/19-induced AMPK and NRF-1 expression [42]. However, further studies are necessary to identify the exact mechanisms involved with SG-induced augmented insulin clearance.

In summary, we confirmed that the SG mice model accurately represents the human surgery outcomes, with SG-induced increased insulin clearance as another parameter to support it. However, one limitation of our study is the absence of a sham-operated body weight-matched control of SG mice. This control group could be important to determine if the increase in insulin clearance occurs due to SG *per se*, an indirect effect of the weight loss, or both. Nevertheless, the increased insulin clearance observed in HFD-SG mice was linked to higher hepatic IDE expression, thus, pointing to SG as a good strategy to counteract hyperinsulinemia in obesity pathology.

## 4. Materials and Methods

### 4.1. Animals

Three-week-old C57BL6/J male mice were obtained from the University of Campinas Facilities and maintained at 22 ± 2 °C on 12 h light-dark cycle. At 4 weeks old, the animals were divided into two groups: the control group (CTL), which received standard chow (Nuvital, Colombo, Brazil), and the high-fat diet group (HFD), which received a diet containing 45% kcal from lipids (Prag Soluções, Jaú, Brazil). At 14 weeks old, HFD mice were divided into two groups where one group was submitted to sleeve gastrectomy (HFD-SG), and the other group underwent a sham operation (HFD-SHAM). Mice were weighed once a week until the end of the experimental period, and the AUC was calculated by trapezoidal integration using GraphPad Prism^®^ (Version 6.00, San Diego, CA, USA). Euthanasia was performed at 20 weeks old. All experiments were approved by the Animal Care Committee at UNICAMP (license number: 5242-1/2019).

### 4.2. Sleeve Gastrectomy and Sham Operations

After 12 h of fasting, mice were anesthetized with 1% isoflurane (BioChimico, Itatiaia, Brazil) with nasotracheal intubation (1 L/min O_2_). For the Sham group, an incision was made in the epigastric midline of the abdomen, the stomach and abdominal cavity were exposed, and the small intestine was massaged using a sterile scalpel handle. Before suturing, a dose of 20 mg/kg of Enrofloxacin (Chemitril^®^, Chemitec^®^, São Paulo, Brazil) and 5 mg/kg of Tramadol (Vitalis^®^, Bogotá, Colombia) was administered to the abdominal cavity. The laparotomy was closed with a continuous suture, with 6-0 polypropylene thread, as well as the skin. For SG mice, an incision was made in the epigastric midline of the abdomen, and the stomach was exposed. The incision was performed from His angle, and 80% of the stomach was removed, including complete resection of the gastric fundus, forming a gastric tube connecting the esophagus to the duodenum [43]. Before suturing, a dose of 20 mg/kg Enrofloxacin (Chemitril^®^, Chemitec^®^, São Paulo, Brazil) and 5 mg/kg Tramadol (Vitalis^®^, Bogotá, Colombia) were administered to the abdominal cavity. The laparotomy was closed with a continuous suture, with 6-0 polypropylene thread, as well as the skin. Mice received 20 mg/kg of Enrofloxacin (Chemitril^®^, Chemitec^®^, São Paulo, Brazil) for 7 days after surgery and 2 mg/kg of Meloxicam (Eurofarma^®^, Itapevi, Brazil) plus 5 mg/kg of Tramadol (Vitalis^®^, Bogotá, Colombia) for 2 days. Mice were kept on a liquid diet for 5 days after surgery. Mice were given doughy HFD beginning on day 6, and on day 12, they returned to solid HFD.

### 4.3. Intraperitoneal Glucose (ipGTT) and Insulin (ipITT) Tolerance Tests

Mice were subjected to 12 h of fasting to perform ipGTT. Blood glucose level was measured with a glucometer (Accu-chek^®^, Roche, Basileia, Switzerland) at times 0, 15, 30, 60, 90, and 120 min after receiving an intraperitoneal glucose dose of 2 g/kg. For ipITT, after 4 h of fasting, the blood glucose level was measured (time 0) by a glucometer (Accu-chek^®^, Roche, Basileia, Switzerland). Mice then received an intraperitoneal administration of insulin of 1 U/kg; thereafter, glycemia was measured at 3, 6, 9, 12, and 15 min. The results were analyzed by calculating the glucose and insulin AUC using the trapezoidal integration in the GraphPad Prism^®^ (Version 6.00, San Diego, CA, USA). Both tests were performed with 14-week-old (preoperative) and 20-week-old (postoperative) mice.

### 4.4. Plasma Insulin and C-Peptide Measurements

Mouse insulin (Catalog #10-1247-1, Mercodia, Sylveniusgatan, Sweden) and C-peptide ELISA Kits (Catalog #EZRMCP2-21K, Millipore, Darmstadt, Germany) were used to measure plasma insulin and C-peptide. Plasma samples were obtained by centrifugation of blood samples at 11,000 rpm for 15 min at 4 °C. The assays were performed as indicated by the kit’s protocol. The blood samples for insulin and C-peptide measurements were collected in fed and fasting states at the end of the treatment. The C-peptide:insulin ratio of the same samples was calculated to quantify the insulin clearance [32].

### 4.5. Western Blot Analysis

Liver samples were collected and homogenized with lysis buffer (10 mM EDTA, 100 mM tris base, 100 mM sodium pyrophosphate, 100 mM sodium fluoride, 10 mM sodium orthovanadate, 2 mM phenylmethylsulfonyl fluoride, 1% Triton X-100, and 1 µg/mL aprotinin). After centrifugation at 12,000 rpm for 30 min (4 °C), the protein was determined using Bradford reagent (BioAgency Biotecnologia, São Paulo, Brazil). For SDS (sodium dodecyl sulfate) polyacrylamide gel electrophoresis, all samples were treated with a Laemmli buffer containing dithiothreitol. After heating at 100 °C for 5 min, proteins (30 µg) were separated by electrophoresis in a 10% polyacrylamide gel. The transfer to nitrocellulose membranes was performed in a Trans-Blot transfer for 2 h in 100 V, with a tris/glycine buffer. After transfer, the membranes were blocked with 5% BSA for 1 h and then were incubated with polyclonal antibodies against CEACAM1 (Catalog #14771; Cell Signaling, Danvers, MA, USA), IDE (Catalog #ab32216; Abcam, Cambridge, UK), and the internal control α-tubulin (Catalog #T5168; Sigma-Aldrich, St Louis, MO, USA). Visualization of specific protein bands was performed by incubating the membranes with appropriate secondary antibodies. Protein bands were visualized using the Amersham Imager 600 (GE Healthcare Life Sciences, Buckinghamshire, UK), which detected chemiluminescence. The band intensities were quantified with ImageJ software (National Institutes of Health, Bethesda, MD, USA).

### 4.6. IDE Activity Measurements

Liver IDE activity was measured using a SensoLyte 520 IDE Activity Assay Kit (Catalog #AS-72231; AnaSpec, Fremont, Canada) by following the manufacturer’s instructions. Total IDE activity was calculated as described previously [15] and normalized per μg of protein content, as determined using a Bio-Rad Protein Assay Dye Reagent Concentrate (Catalog #5000006, BioRad, Hercules, CA, USA).

### 4.7. Statistical Analysis

The data are presented as the mean ± standard error of the mean (SEM). To evaluate data normality, we applied the Shapiro–Wilk test. When normal, we used the parametric Student’s *t*-test (to compare two groups) or a one-way ANOVA with an unpaired Tukey’s post-hoc test (to compare three groups); otherwise, the non-parametric Mann–Whitney test (to compare two groups) or Kruskal–Wallis test (to compare three groups) were adopted. The difference between groups was considered statistically significant if *p* ≤ 0.05.

## Data Availability

Data are contained within the article.
